# Modules and Techniques for Motion Planning: An Industrial Perspective

**DOI:** 10.3390/s21020420

**Published:** 2021-01-09

**Authors:** Stefano Quer, Luz Garcia

**Affiliations:** 1 Department of Control and Computer Engineering, Politecnico di Torino, Corso Duca degli Abruzzi 24, I-10129 Turin, Italy; 2 Automated Driving Technologies, Technology Innovation, Marelli Europe S.p.A., Viale Carlo Emanuele II 150, Venaria Reale, I-10078 Turin, Italy; luz.garcia@marelli.com

**Keywords:** path planning, autonomous driving, sensor data and map manipulation, many-core architectures, software engineering

## Abstract

Research on autonomous cars has become one of the main research paths in the automotive industry, with many critical issues that remain to be explored while considering the overall methodology and its practical applicability. In this paper, we present an industrial experience in which we build a complete autonomous driving system, from the sensor units to the car control equipment, and we describe its adoption and testing phase on the field. We report how we organize data fusion and map manipulation to represent the required reality. We focus on the communication and synchronization issues between the data-fusion device and the path-planner, between the CPU and the GPU units, and among different CUDA kernels implementing the core local planner module. In these frameworks, we propose simple representation strategies and approximation techniques which guarantee almost no penalty in terms of accuracy and large savings in terms of memory occupation and memory transfer times. We show how we adopt a recent implementation on parallel many-core devices, such as CUDA-based GPGPU, to reduce the computational burden of rapidly exploring random trees to explore the state space along with a given reference path. We report on our use of the controller and the vehicle simulator. We run experiments on several real scenarios, and we report the paths generated with the different settings, with their relative errors and computation times. We prove that our approach can generate reasonable paths on a multitude of standard maneuvers in real time.

## 1. Introduction

Several sources [1,2] report that more than 90% of road accidents are caused by human error, with economic and social costs of several hundred billion US dollars. Automated Driving Systems (ADSs) are becoming extremely popular in modern societies. Expected benefits range from the promise of reducing accidents due to human error to decreasing transportation costs, lowering emissions and environmental impact, facilitating movements of mobility-impaired people, and lessening driving-related stress. However, autonomous systems might not eliminate all accidents and may even perform worse than a human being in nasty circumstances due to complex driving environments or bad weather. Unfortunately, as stated by Blanco et al. [3], today we have not enough data to make a statistically significant evaluation of autonomous driving. Kalra et al. [4] evaluated that it would take approximately 5 billion miles to demonstrate that the failure rate is below some specified threshold. They also computed that with a fleet of 100 autonomous vehicles, driven 24 h a day, 365 days a year, and at an average speed of 25 miles per hour, it would take about 225 years to travel this distance.

Current ADSs must perform many tasks. An autonomous car is a terrestrial driver-less vehicle that is able to sense its surrounding, to move with little or no human intervention following a given path, to avoid all possible obstacles, and to respect traffic rules. Thus, modern systems are becoming increasingly complex, and, consequently, difficult to test. Following [5], autonomy driving is characterized by the amount of autonomy level of the vehicle. This parameter is defined within 6 levels of autonomy, ranging from no driving automation (level 0) to full driving automation (level 5). Although many of the technological components required for a fully autonomous vehicle are already in place today, level 5 vehicles are probably several years away.

Since the autonomous car is a type of robot, different techniques have been studied in distinct domains, and different approaches have been considered over the years. This work focuses on an industrial experience made by the Automated Driving Technologies group at Marelli in Italy in cooperation with the Department of Control and Computer Engineering at Politecnico di Torino in Italy. The target of the project was to build and test a fully working ADS using components partially selected among the ones available on the market and partially redesigned from scratch. Among the main contributions, we will focus on the work done to implement some of the custom-made components. At the same time, a consistent part of the work consisted of integrating these home-made modules with all others off-the-shelf components. Thus, one of the core parts of our task was to represent and communicate information efficiently between the different modules of our system. For example, environment maps are usually quite memory expensive and the cost to represent and transfer them between different units is not negligible. We will show how a synchronous communication strategy, between the data fusion module and the path planning unit, reduces computational costs and memory usage but it leads to global behaviors that are not reactive enough in critical situations. Thus, we will suggest an asynchronous communication scheme, in which the data-fusion module must prepare more information for the path planner during every working cycle. This approach will imply higher computational and transfer costs but more reactive behaviors. Similar considerations can be made when the modules involved during the communication are actually different computational units within the same module, such as the CPU and the GPU, or even different kernels within the GPU-based path-planner implementation. Moreover, great effort was dedicated to test and improve the final system using a simulator with the initial versions and then on a on-board system directly on the road. Overall, that the target was not only to test the final performance of the system but also to evaluate the effort, time, and cost required to complete it.

### Our System

**Figure 1:**
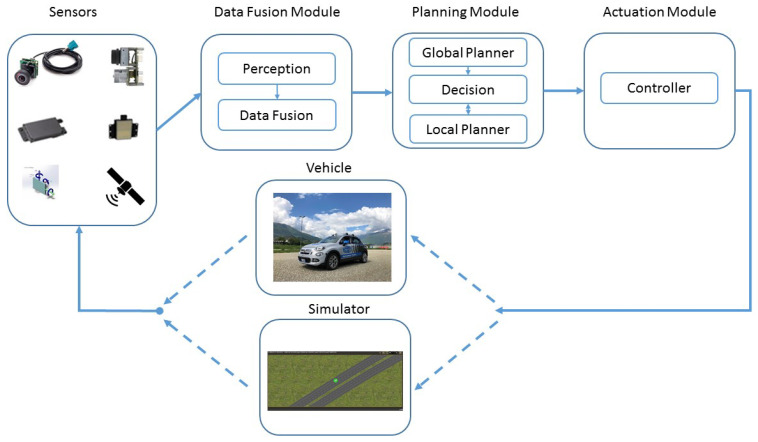
The high-level structure of our ADS. The sensor system, made up of about 30 different sensors, collects adequate information about the vehicle’s surrounding area in real time. Starting from this information, the data-fusion unit builds occupancy grid maps, and it detects the free areas around the vehicle. Given this environmental information, the planner generates the collision-free trajectories the vehicle is supposed to follow. Based on the selected path, the controller generates commands for the vehicle, and it verifies its dynamic behavior. Finally, a real car (or a vehicle simulator) follows the controller’s commands, and it closes the ADS loop chain.

In our environment, the path planning is structured as shown in Figure 1, and it includes the sensor units and the data-fusion system, the path planner, and the vehicle controller. A simulator (during the first stages) and a real vehicle (when more advanced configurations are available) are used to test the overall result.

The data-fusion system receives data from the sensor devices and it generates pre-processed data, such as maps of the environment with information about the obstacles, the vehicle constraints, its speed, its position, and so on. This information must be transferred to the planning module, and, once within the path planner, between the CPU and the GPU units, or among the different CUDA kernels which implement the local planner. Maps have high memory requirements, they should be precise, and they should enable the planner to make good guesses about the future. In this work, we specify which are the time constraints of the entire system and we highlight the main problems we encountered to transfer those maps in raw form. Moreover, as many moving objects (such as pedestrians) have an erratic behavior, it is a matter of discussion to decide how much the internal representation must be fined-grained and precise. To this extent, we suggest some strategies to reduce image size requirements and transmission costs, and we experiment with different map transmission rates.

Within the planner module, the global planner, driven by the user’s commands, generates long-term trajectories. Then, the decision-maker produces short-term maneuvers. Finally, the local planner transforms these maneuvers into the final trajectory the car is supposed to follow. To reach this final target, the local planner usually generates several admissible paths and it selects the best one among them. The selected trajectory is supposed to follow a reference path, and it must be, from the one hand, cost-effective and suitable for the vehicle, and, from the other one, collision-free and safe. To satisfy these constraints, Schwesinger et al. [6] adopted *Rapidly exploring Random Trees*. The key idea of this approach is to generate a tree-like set of paths, and to use such a tree to explore the state space along with a given reference path. A proper cost function is finally used to select the best path among the ones represented by the tree. This strategy proved to be efficient to navigate complex environments including static and dynamic obstacles. It also easily takes into consideration the dynamics of the vehicle and the shape of the surface. At the same time, it has been proved to be quite expensive from the computational point of view, motivating the adoption of GPU (Graphical Processing Units) to re-engineering complex algorithmic phases [7]. The idea of this approach is to optimize all main computational steps, and to improve the efficiency and scalability of the method, using a massively parallel implementation. In the current paper, we enclose the stand-alone local planner presented by Cabodi et al. [7] within our complete environment, in which all sub-systems and aspects of the methodology (such as the map generation, the communication times, etc.) are properly taken into consideration.

The trajectories generated by the planner, are fed to a vehicle controller, which transforms these trajectories into suitable commands for the vehicle or an “equivalent” simulator. The vehicle’s status influences the sensors’ perception, closing the loop represented in Figure 1.

A simulator or a real vehicle are usually adopted to verify the overall results and the system reactivity. In our case, and our experimental section, we report and analyze data collected on the field. We concentrate on standard maneuvers on which we compare several possible configurations either based on CPU-based computational units or many-core concurrent GPU-based ones. We also analyze several critical aspects of the original algorithm, and we present the impact that our setups may have on real-world scenarios and the paths suggested by the application.

All modules represented in Figure 1 will be analyzed in the following sections. The data-fusion module is the target of Section 3. Section 4 concentrates on the path-planner module. Section 5 focuses on the controller and the vehicle simulator. Section 6 includes considerations on the communication protocol used to transfer information among the previous units. Section 7 concludes the paper with some summarizing experimental results. Finally, Section 8 draws some conclusion derived from this experience.

## 2. Related Works

Even if it is possible to find projects on autonomous vehicles since 1984, the DARPA Grand challenges [8,9], held for the first time in 2004 in the Mojave Desert region of the United States, drastically contributed to increasing the interest toward this technology. Thus, huge investments by several automotive industries, and associated companies (such as Google), have been done in recent years. Many technical aspects of automated driving have been recently discussed by Huang et al. [10] and by Yurtsever et al. [11]. Other works, such as the one by Grigorescu et al. [12], survey the use of specific strategies in self-driving architectures, such as convolutional neural network, recurrent neural network, and deep reinforcement learning. Consequently, due to the enormous literature in the field, we will mainly review the papers more directly related to the modules to which we dedicate more attention in the following sections.

Object tracking has been first performed using particle filters. Valiska et al. [13] compare particle filter object tracking using a single color with a filter that uses observation of object color through color histograms. Yildirim et al. [14] integrate the color distribution of objects into particle filtering to track moving vehicles with temporal disappearance. Qing et al. [15] use a particle filter to overcome the challenge of multiple vehicle tracking with partial vehicle occlusion and temporarily missing vehicle sections. Chan et al. [16] generate the probability distribution of vehicle under particle filter framework to detect preceding vehicles on the highway under various lighting and different weather conditions. More recently, object detection has been faced with deep learning strategies. The works by Zhao et al. [17] and Wu et al. [18], review recent deep learning-based approaches to perform object detection. A first family of techniques, i.e., two-stage detectors, are described in works such as the one by Girshic et al. [19] on region-based methods. A second family of strategies, i.e., one-stage detectors, are described by Redmonn et al. [20] with YOLO and its variants. Convolutional neural networks have also been used to solve the problem of high-resolution aerial scene classification, as the one presented by Petrovska et al. [21].

Several works have been focused on grid-map representation and its optimization. Matthaei et al. [22] present a grid-based approach to detect road lanes in urban environments, with no lane markings, which is robust against a wide variety of urban scenarios. Kretzschmar et al. [23,24] address the problem of efficient, information-theoretic compression of pose graphs. Approaches are based on removing sensor information from the map without losing too much information, or on maintaining the same map unless previously unobserved parts of the environment are explored.

As local trajectories are subject to stringent constraints and imply intensive computations, path planning modules have been developed adopting different frameworks. Solea et al. [25] adopt geometric planning techniques, developed within a mobile robot framework, to transform the planning problem into an interpolation task. Albeit these methods have low computational costs, they proved to be inappropriate for real applications, as the computed trajectories are often neither feasible nor optimal. Schwesinger et al. [6] use deterministic sample-based algorithms, based on powerful numerical forward propagation and sampling methods, to rapidly find correct trajectories Werling et al. [26] use control strategies to express the planning process, through differential equations, as a nonlinear programming task. Unfortunately, this notation hides severe problems as they rely on numerical methods which are often trapped in local minima, are time-consuming, and are thus unsuitable for real-time applications. Sample-based planning techniques [6,27], sample the configuration space into a set of discrete motion goals and do not need sophisticated mathematical approaches. Randomized algorithms are mostly based on Rapidly exploring Random Tree [28].

Some path planning framework has also recently been based on GPU computations [29,30,31,32]. Generically speaking, these works introduce motion planning algorithms that exploit the high computational capabilities of many-core GPUs to efficiently perform expensive computations. For example, Kinder et al. [30] implement a randomized version of the $A^{*}$ algorithm in which the search is performed by a CUDA kernel.

As far as ADS testing is concerned, Huang et al. [33] review works on autonomous testing, including autonomous vehicle functional verification and system validation. Chen et al. [34] propose a new simulation platform with hardware-in-the-loop. Their platform, made up of four layers, presents multiple new features going from the ability to simulate various sensors and virtual testing fields, to the capacity to performs a closed-loop evaluation of the scene perception and the entire planning platform. Moreover, it also enables rapid migrations of control and decision-making algorithms from the virtual environment to real self-driving cars.

## 3. Sensors, Perception and Data Fusion

This section focuses on the sensors (their selection and disposition inside the car), the data-fusion module, and the communication and synchronization between the data fusion and the path planning module.

### 3.1. Sensors and Perception

**Figure 2:**
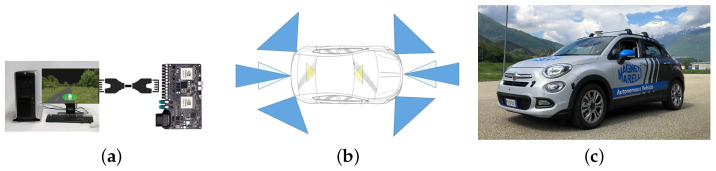
(**a**) The data fusion, planning and actuation module running on the PX2 NVIDIA GPU PG418 board and a desktop (or a laptop). (**b**) Sensors’ activity around the vehicle. (**c**) The final car configuration with on-board hidden sensors.

To properly react to any situation, it is crucial to acquire adequate information about the vehicle’s surroundings in real time. With challenges such as DARPA in 2004, the interest toward autonomous driving increases dramatically, and this interest has brought innovation on the sensor side as well. Figure 2a shows our system running on a PX2 board feed with virtual sensors generated and running on a virtual environment, Figure 2b the sensors’ activity around the vehicle, and Figure 2c the final vehicle used to perform all testing.

In most of the cases, researchers focused on gathering information from one or more cameras mounted on the moving vehicle. Unfortunately, this may be highly insufficient in modern path planning and security systems. As demonstrated by Kocic et al. [35] and other researchers [36] including more sensor devices into the system makes systems more reliable, robust and it improves its performance. In our system, we can divide sensors in the following 4 main categories:

- In the first group, we insert video-cameras. The cameras on the market can produce from 30 to 60 frames per second, and they can easily identify obstacles, relevant traffic signs, and appropriate navigation paths. Common off-the-shelf cameras have low cost, and their output can be manipulated by several applications using computer vision algorithms and/or machine/deep learning approaches [12,37]. Unfortunately, cameras can perform badly in nasty environmental conditions: It is difficult to evaluate distances in rainy conditions, to track lanes in foggy weather, and to discriminate between objects and dark shadows in sunny conditions. For that reasons, using other sensors is in order.
- In the second category, we insert radars. Radars working at a frequency rate from 24 GHz to 76 GHz have a spanning range varying from short to medium, and are thus useless for far-away object detection. Luckily, with the advent of the so-called “long-range radars”, working at 77 GHz, it is now possible to increase ranges up to 200 m and far-away objects do not constitute a problem anymore. Moreover, radars work well in all weather conditions, and the information collected does not degrade with bad weather. Unfortunately, radars have a low resolution, and it is often difficult to discriminate between true and false positive.
- In the third category, we insert LiDARs (Light Detection and Ranging). Lidars are a way for measuring distances by illuminating the target with laser light and evaluating the reflection with a sensor. They produce high-resolution point clouds and quite reliable information thus they deliver good distance estimations and are primarily used for roadside detection (including lane detection), recognition of both static and dynamic objects, Simultaneous Localization and Mapping (SLAM), and 3D environmental map creation. Unfortunately, they may be very expensive. To reduce the cost factor especially on inexpensive cars, Marelli in January 2020 has signed an agreement to develop more practical LiDAR solution for automotive applications, in collaboration with XenomatiX.
- In the fourth category, we insert high precision GPS and IMU (Inertial Measurement Unit) which are needed within the vehicle positioning system. They are fundamental for an autonomous car, because they give the knowledge of the vehicle location.

Using these 4 sources of information, each vehicle is potentially capable of generating a map of the surrounding environment. Anyway, the final sensor configuration strongly depends on the operation condition. Following the Operational Design Domain (ODD) model [38], any final sensor configuration should accurately take into consideration the final vehicle application. For example, urban autonomous driving requires more accurate short-range sensoring, whereas for highway autonomous driving long-range sensoring is more important. To satisfy, different requirements, and guarantee a broadly usable path planning system. Marelli chose the following sensor configuration: 2 long-range radars, 4 medium-range radars, 8 LiDARs, and 14 cameras.

### 3.2. Data Fusion

Research on mobile robot navigation has produced two major paradigms for mapping environments, i.e., topological-based and grid-based [39]. As topological maps are considerably difficult to learn in large-scale environments, we resort to grid-based representation. The basic idea of an occupancy grid is to represent a map of the environment through a Cartesian coordinate system. The map consists of a matrix of evenly spaced locations, where each location corresponds to a square region on the map. For each location, a probability value is represented, indicating the probability of the presence of an obstacle in that specific location. The precision of the map varies: If more digits are added to a grid reference (or to the probability value) then the reference becomes more accurate. Unfortunately, at the same time, the quantity of memory required to store the grid increases.

More precisely, if we let m[x][y] denote the grid cell with indices *x* and *y* (in a 2D map, the two indices are used to represent the two dimensions), then the notation p(m[x][y]) represents the *posteriori probability* that cell [x][y] is occupied. Since the intrinsic geometry of a grid corresponds directly to the geometry of the environment, the vehicle’s position within its model can be determined by its position and orientation in the real world. Consequently, different positions for which sensors measure the same values (i.e., situations that look alike) are naturally disambiguated in grid-based approaches (which is not the case for topological approaches).

To build occupancy grid maps and to detect free space, we follow the state-of-the-art approach proposed by Li et al. [40]. First, using an artificial neural network adopting back-propagation, we translate sensor reading into occupancy values. The input of the network consists of the four sensor readings closest to m[x][y], along with two values that encode m[x][y]. The output of the network is the value of p(m[x][y]). The interpretation of the sensors is integrated over time, to yield a single, consistent map. Based on the Bayes’ theorem, the new data in the current measurement cycle is combined with the previous data during the mapping of occupancy grid, to calculate the posteriori probability p(m[x][y]) over the entire map given the data. With a forward inverse sensor model, the reflection data from sensors are converted into the probability, which is then used as the detection probability in the Bayes’ theorem. If the sensors detect an object, the grid is recognized as occupied where the target is located. The free space is defined as a linear function of the distance between the sensors (the vehicle) and the target. The grids without any measurement information are marked as unknown.

### 3.3. Data Compression and Memory Optimization

Grid maps must be transferred to the path planner and they should be updated with a high frequency, as a high refresh rate guarantees a higher safety. As the data-fusion module and the path planner do not share any memory, a critical aspect of the system is how to organize the communication between them. As analyzed in Section 2, several works have been proposed to reduce the memory cost to represent grid maps. As we must target both memory optimization and fast map manipulation, we worked in the following directions.

**Figure 3:**
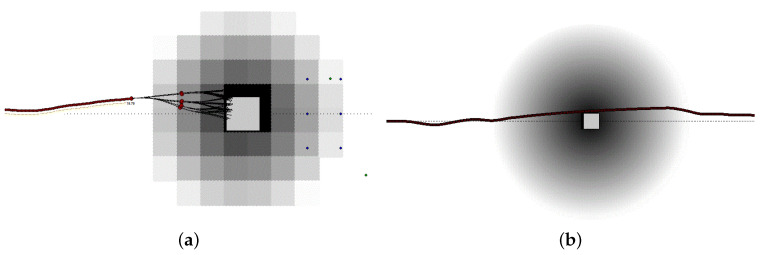
Trading-off between the resolution and the memory requirement of a map: The spatial resolution can be reduced to decrease the image size, but the counter-effect is to represent objects too blurred to be correctly identified. The figure shows a path colliding with an object (represented with a white color). Gray colors represent the level of danger, i.e., the darker the tone the higher the danger. In (**a**), the map resolution is equal to (3 m × 3 m) per pixel and the obstacle is represented at a distance of 30 m. The object is definitely too blurred to be appropriately identified. Thus, in (**b**), the map resolution has been increased to (0.5 m × 0.5 m) per pixel and the obstacle is represented at distance of 60 m.

To reduce the memory requirements, we first play with spatial resolution. To focus on real data, each grid map created by our fusion system corresponds to an image of (1000×1000) pixels. Each pixel, which represents the posteriori probability for the corresponding position to be occupied, may be represented with a data type ranging from a short integer of 1 byte to a standard floating point of 4 bytes. Thus, memory requirement varies from 1 to 4 MBytes for each map. To reduce this value, we performed the following analysis. Figure 3a represents a map with a low spatial resolution, as each pixel represents a square area of size (3 m × 3 m) on the ground. In this configuration, each obstacle proved to be too coarse, already at a distance of 30 m, to accurately evaluate the quality of the path. Figure 3b represents a map with a higher resolution, as each pixel represents (0.50 m × 0.50 m), and the entire map represents an area of (500 m × 500 m). Considering that at a speed of 60 m/s (i.e., 216 Km/h) a vehicle travels 180 m in 3 s, this area may be considered even larger than required. Consequently, we decided to reduce the map resolution down to (500 × 500) pixels, and possibly to relocate the car within the map to represent a larger space in front of the car and a smaller space behind it. Thus, given the original image resolution, we crop it to (500 × 500) pixels, and we maintain 400 pixels before the vehicle, and only 100 behind it. This process cuts down the memory occupation to 25% of the original one, but it maintains 80% of the original spatial acuity before the car.

To store our maps as efficiently as possible, we decided to use fast GPU texture memories to store them. Following Doggett [41] individual elements in the texture map are called texels (from “TEXture ELements”) to differentiate them from the screen pixels. In one texture *T*, every texel t∈T is represented using the 4 RGBA channels (R, G, B and A). In this way, each pixel is represented on 4 floating point values (x,y,z,w), thus, we can encode from 4 to 16 occupancy grid-map pixels *p* into one texel of the texture *T*. This encoding also reduces the number of memory accesses performed by the GPU, as one single access (read or write) on a RGBA field may manipulate from 4 to 16 pixels. Obviously, there is a trade-off between the two possibilities, as is we reduce the memory occupation, we need more time to encode and decode the data related to each pixel. Experimentally, we discovered that it is sufficient to represent the probability on a single byte, which guarantees 256 different levels. We diagrammatically represent this situation using gray tones, as in Figure 3, where the real object is white and gray colors represent the level of danger, i.e., the darker the tone the higher the danger. Then, we map up to 16 picture pixels on a texture texel. We resort to the simplified storing strategy, i.e., 4 picture pixels into a single texture texel, only when time constraints become really tight due to an extremely high number of computations the GPU must perform in one single cycle.

**Figure 4:**
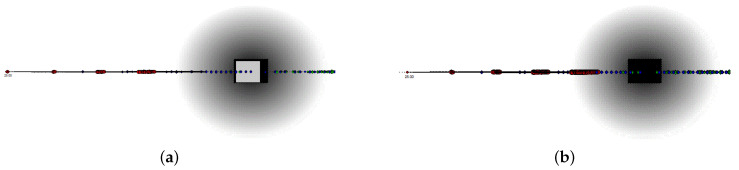
Improving safety by object enlargement while predicting the object location. An obstacle, represented at a distance of about 30 m, is moving on the vehicle path in the same direction of the vehicle. (**a**) represents the position of the object at time $T=T_{0}$ in white, and its position after 20 ms, i.e., at time $T=T_{0}+20$ ms, in black. As the object travels only 0.8m between the two frames, we represent all intermediate locations as occupied. (**b**) shows the corresponding grid map generated by our system. Gray tones are used to represent the different probability of the object of being in each location, i.e., when the tone is darker the danger for the vehicle is higher.

Memory requirements and communication costs can also be reduced working on the prediction model. Objects are tracked over time. Static objects are detected at the same location during several cycles; thus, for static object, a stable occupancy grid map may be achieved and hence the noise, and the uncertainty, of the measure are eliminated. On the contrary, the position of dynamically moving objects changes, forcing the system to forecast their future location. This step is of paramount importance to take responsible decisions during planning [42,43]. As we will better analyze in Section 4, our path planner runs every 20 ms and it works with a look ahead time of 3 s. An object moving at 3 m/s (about 11 km/h) travel 0.06 m in 20 ms, whereas an object moving at 40 m/s (144 km/h) travels 0.8 m in the same time. As these values are small enough and any prediction far away is the future becomes inaccurate, we estimate the position of each object during a specified number of time frames in the future. Then, we represent all these locations as occupied, with different probabilities, into every grid map within the map set representing these time frames. This can be done by computing the probability p(m[x][y]) of each location as the average value of the probabilities of the same location during all considered time frames. As an example of this process, Figure 4 shows a grid map representing an object moving along the horizontal axis (from left to right) with a speed of 5 m/s (18 Km/h). Figure 4b shows the final grid map with the probability of each location to be occupied in the future represented by gray tones.

## 4. The Path-Planner Module

The path-planner module is the key to produce fully autonomous vehicles. The path planner completes three main tasks: *Mission planning*, i.e., the vehicle solves a routing problem in order to complete a given task; *decision-making*, i.e., the vehicle chooses an appropriate action for the next time step from an available action set; *path planning*, i.e., the vehicle plans its future trajectory as a function of space or time [44]. Consequently, its main goal is to generate a collision-free and safe path, bringing the vehicle to its destination, and taking into account the vehicle dynamics, its maneuver capabilities in the presence of obstacles, alongside with traffic rules and road boundaries. As shown in Figure 1, the path planner includes the global planner, the decision-maker, and the local planner, which we will describe in the next subsections.

### 4.1. Global Planner

**Figure 5:**
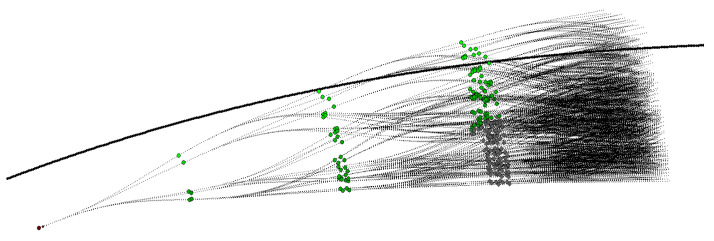
A random tree exploring the area around a given curved trajectory. The degree of the tree *D* and its height *H* are equal to 4. The car is initially placed at the center of the lane and its target is to converge on the specified path (black line). To have an idea of the convergence speed, the lane is 6 m wide and the trajectory about 40 m long.

The global planner is in charge of finding an optimal path given the current position of the vehicle, its final destination, and a proper knowledge of the environment and the position of all obstacles along the way. As discussed by Marin-Plaza et al. [45] several methods have been used over the years to analyze maps and to obtain the reference path. Some of these methods are based on Voronoi diagrams. Others focus on search algorithms as Dijkstra, Best First and A*. Recently, the research community is centering its efforts on rapidly exploring random trees and the use of neural networks [46]. We combine these two approaches to obtain a reference path (i.e., the black line in Figure 5) coherent with the actual environment. Our global planner extracts the road network information from stored maps and it combines this information with data coming from the grid maps generated by the data-fusion system described in Section 3.

### 4.2. Decision-Maker

As discussed by Mirchevsa et al. [47] the first high-level decision-making algorithms were based on building a system of rules and deducing the optimal maneuver at run-time. The disadvantages of this strategy become obvious when the methodology is applied to traffic scenarios with increasing complexity. In complex situations, it is necessary to manage corner cases, and this necessity requires the addition of new rules to the existing rule-based system. Usually, this process vastly increases the complexity of final the decision-making procedure, and it makes the designing and development phase harder and longer. For this reason, many recent works rely on decision-makers based on machine learning strategies [44,47,48]. The core feature of machine learning approaches is to improve the applied strategy over time, based on the previous experience. In this framework, Reinforcement Learning often adopts an initial representation of the environment, which is very simple, but sufficient to apply the method. Starting from this simple representation, many complex tasks can be learned based on the interaction between the vehicle and the environment. Simple working frameworks can drastically accelerate the learning process, reducing the time-to-market. Moreover, Reinforcement Learning can be applied to large-scale systems with infinite state and action spaces. Unfortunately, safety is of paramount importance in any condition, and especially when the system learns new activities in real traffic conditions, with many other traffic actors. In these situations, avoiding collisions at all costs, and performing only safe maneuvers, increases the complexity of the design phase and the time required to conclude it. Moreover, the best reinforcement learning algorithms are not transparent today, and this is a strong limitation for their applicability on a large scale.

To trade-off between the advantages and disadvantages of the previous methods, we combine them, adopting the generality of reinforcement learning and the transparency of rule-based approaches. Our decision-maker algorithm is massively built and tested with real-time simulations in real-world scenarios and it performs quickly and with low memory consumption.

### 4.3. Local Planner

Local planners based on random trees [6] explore the state space by rapidly generating a set of paths along a given reference path. This set is organized as a tree, represented as in Figure 5, and built, during each run of the planning cycle, on a level-by-level basis. Our local planner is implemented as described by Cabodi et al. [7]. For that reason, this section is not part of our contribution, but it is required to properly understand the synchronous and asynchronous communication schemes presented in Section 6. The main difference between the implementation presented by Cabodi et al. [7] and the current one is that the system now runs on a PX2 NVIDIA pascal GPU PG418 with up to 3 GHz and 8.00 GByte DRAM instead that on a GPGPU NVIDIA GEFORCE GTX 970 with 1664 Cores and 4.00 GByte of memory. In this section, we just report the details necessary to understand Section 6.

At each level, our planner tries to explore (reach) a larger set of objectives starting from the current set of possible vehicle positions. Although the initial position (for the first tree level) coincides with the initial vehicle coordinates, each new objective is computed using a predefined (node or) path splitting policy. Objectives are found guessing the desired vehicle position after $T_{lookahead}$ time units, considering differing vehicle lateral orientation θ and vehicle longitudinal speeds *v*. Each tree level spans the space for $T_{sim}=\left(T_{lookahead}/H\right)$ time units, where *H* is the tree height. At tree depth h=0 the current position is unique and the number of objectives is equal to the number of children *D* of the tree root. At depth h=1, there are *D* current positions and $D^{2}$ targets, etc. The entire process is repeated *H* times, with h=[1,H], generating a tree with *H* levels. The algorithm finally applies a cost function to all trajectories. This function selects the best trajectory according to the given criteria. As it is not guaranteed that objectives are indeed reached, the outcome is the closest feasible tree node, and the corresponding edge, leading from the tree root to the best first level node.

### 4.4. Expansion Trees and Grid Maps

**Figure 6:**
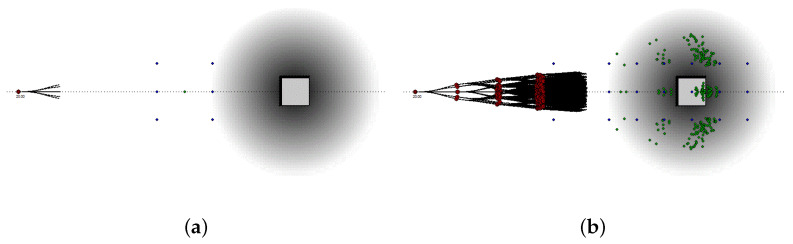
Obstacle avoidance example 1: Static object. The object is detected at the same location during several path-planner cycles. (**a**) reports the expansion tree with height h=1. (**b**) indicates the expansion tree with height h=4. The small green points represent the trajectory targets during the expansion of the rapidly exploring random tree at different tree depth.

**Figure 7:**
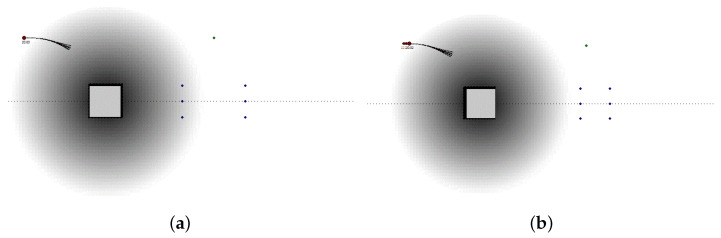
Obstacle avoidance example 2: Moving object represented in contiguous time frames. The two grid maps (**a**,**b**) belong to two consecutive path-planner runs. Figure 7a represent the environment at $T=T_{0}$, and Figure 7b at $T=T_{0}+20$ ms.

**Figure 8:**
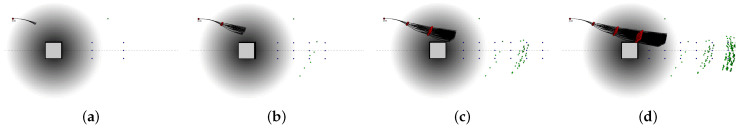
Obstacle avoidance example 3: Moving object represented in distant time frames. The grid maps, and the expansion trees, belong to the same set of maps. The grid maps represent the environment at time $T=T_{0}+\left(h-1\right)\cdot 600$ ms, with the height of the expansion tree equal to h∈[1,4], i.e., $T=T_{0}$ for (**a**), $T=T_{0}+600$ ms for (**b**), $T=T_{0}+2\cdot 600$ ms for (**c**), and $T=T_{0}+3\cdot 600$ ms for (**d**).

The relationship between grid maps and expansion trees is illustrated by the following examples. Figure 6, Figure 7 and Figure 8 show two different scenarios, in which we report a grid maps with the global path hitting an obstacle and the corresponding expansion trees generated to follow the path and to avoid the obstacle.

In Figure 6 the obstacle is statically lying directly on the vehicle straight path. The object is thus detected at the same location during several path-planner cycles. Figure 6a shows the initial expansion tree for h=1, and Figure 6b the expansion tree for h=4. The dots in the pictures show the path planner targets with a degree of the tree equal to D=6.

In Figure 7 and Figure 8 the object is moving along the horizontal axis (from left to right) with a speed of 5 m/s (18 km/h). As the object is moving, the system must forecast its location in different timeframes in the future. In Figure 7 the two grid maps represent the space around the car at time $T=T_{0}=0$ ms and $T=T_{0}+20$ ms. These maps belong to two consecutive path-planner runs, i.e., they are used two generate the same level (h=1) of two different but consecutive rapidly exploring random trees. As in 20 ms, the obstacle travels only at a distance of 0.1 m, the obstacle displacement is undetectable, and the dark area is a good approximation of the obstacle position in both time frames.

Figure 8 shows four grid maps taken at a distance equal to 600 ms. As better analyzes in Section 6, these maps belong to the same path-planner runs, i.e., they are used two generate different levels of the same rapidly exploring random tree. In this scenario, the object travel at a distance of 3 m from one map to the following one. Consequently, we use different grid maps to perform the planning to avoid too large approximation errors by estimating the reality using only one single grid map. In Figure 8, the 4 pictures show the expansion tree with height h∈[1,4].

## 5. Controller and Vehicle Simulator

In this section, we describe the role of the controller and of our vehicle simulator (mainly used during the initial design steps), inside our system.

### 5.1. Controller

With reference to the architecture presented in Figure 1, the controller is the component dedicated to control the dynamic behavior of the vehicle. Given the information computed by the local planner, the controller generates commands to the vehicle. To perform this operation, the controller uses a typical hierarchical structure consisting of an upper-level controller and a lower-level controller. The first one, given the sensor system information, provides reference acceleration and speed for the controlled vehicle; the second one provides low-level actions to manipulate throttle and brake actuators. Over the years, different techniques have been used to manipulate the throttle and the brake, in the literature we can found a variety of examples. Alonso et al. [49] uses a self-tuning PID controller to improve the stability of the system in the presence of noise signals. Lin et al. [50] adopt the Model Predictive Control (MPC) given its very good results in terms of safety and comfort for the passenger.

For the current application, we adopted a Model Reference Adaptive Control (MRAC) technique, ensuring a very good speed tracking during the acceleration and deceleration phase. We also use the novel approach introduced by Trotta et al. [51] and Raffin et al. [52] to compute the safety distance.

### 5.2. Vehicle Simulator

**Figure 9:**
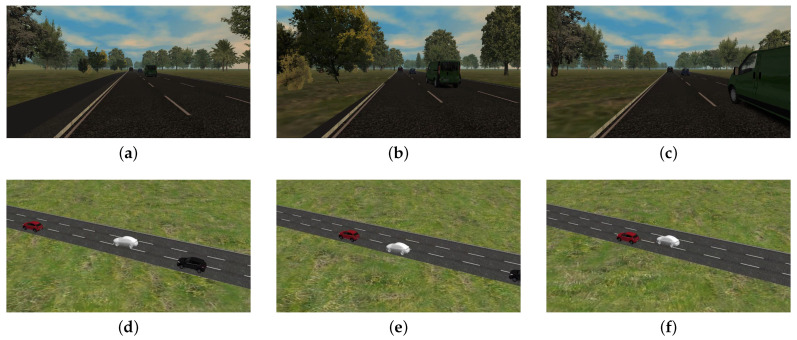
Three image frames extracted from two simulator video-outcomes. (**a**–**c**) show the simulator inner (or driver) view, when the automatic pilot is driving on a highway with medium traffic conditions. (**d**–**f**) illustrate the simulator top (or virtual) view, when the vehicle is executing a double overtaking maneuver.

The two main testing approaches are based on real vehicles and simulators. We use a simulator during all phases of the design. Figure 9 reports a few timeframes of our simulator video-output in two different scenarios and with two different simulation settings. Figure 9a–c show the simulator inner (or driver) view of a highway, while an automatic pilot is driving in medium traffic conditions. Figure 9d–f illustrate the simulator top (or virtual) view of a double overtaking maneuver on a highway, with the adaptive cruise control activated.

Simulation was completed by real vehicle runs in all more advanced and stable configurations of the system. Real-world testing delivers a high simulation accuracy, but it is very time-consuming, expensive, risky, and limited by climate, weather, and scenarios [53]. For real vehicle testing, we use the sensor and car configuration described in Section 3.1 and Figure 2.

**Figure 10:**
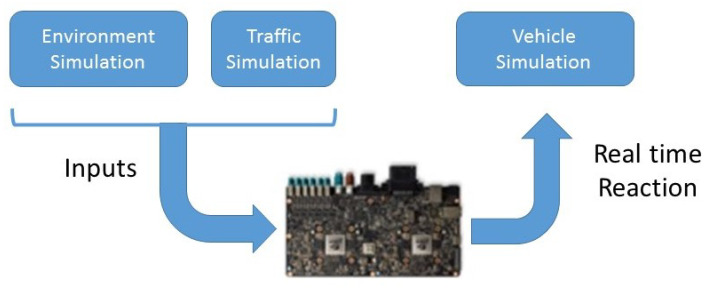
HIL (Hardware-in-the-loop) simulation configuration.

As far as simulation is concerned, simulation testing requires a virtual environment which implies writing accurate enough software to model a complete driving scenario. This software should include the virtual driver, all sensors, the surrounding environment, the traffic conditions, and realistic vehicle dynamics. Once all this is done, in contrast with real-world testing, virtual environment simulation is safe, repeatable, and controllable. Moreover, it allows testing self-driving cars in various scenarios helping to validate many aspects of a vehicle at a time, decreasing development costs whenever possible. We followed the Hardware-in-the-loop (HIL) approach, where the target hardware of the vehicle that runs the autonomous driving software stack is directly stimulated by the scenarios running on the simulation environment. Our HIL is represented in Figure 10. With this approach, it is possible to test and validate the same software and hardware system that will operate on the autonomous vehicle. As discussed by Chen et al. [34] and by Huang et al. [33] using the HIL, and connecting the PX2 NVIDIA Electronic Control Board (ECU) and the simulation interface in close-loop, significantly enhance the efficiency of the core algorithms. Furthermore, applied to our system, as represented in Figure 1, this approach enables us to:

- Test the range, accuracy, and tracking capabilities of our environment sensors.
- Check the ability of our fusion system to re-create the environment.
- Verify the trajectory against obstacles and other vehicles.
- Testing the vehicle stability and control accuracy on the control module.

Even if Marelli has developed internally the software interface between the ECU and the virtual environment, for this project, we use CarSim for vehicle dynamics and SCANeR studio for traffic management, surrounding environment, and virtual sensors in co-simulation.

## 6. Communication Schemes

Giving the framework of Figure 1 and the devices described in the previous sections, one of the main issues is to establish a proper communication scheme among the modules. Mover specifically, we must transfer maps between different logic units and different CUDA kernels running within the GPU. To describe, our communication protocol, we suppose that the fusion system generates a new grid maps every $T_{g}$ (*Generation Time*) seconds. Similarly, we indicate with $T_{cycle}$ (*Cycle Time* or *Planning Time*) the time required by the local planner to compute a new path. $T_{cycle}$ represents the main temporization constraint for the entire system and it strongly influences its performance. Depending on the values of $T_{g}$ and $T_{cycle}$, we may organize the data transfer between the data fusion an the path-planner module synchronously or asynchronously.

### 6.1. Synchronous Path-Planner

**Figure 11:**
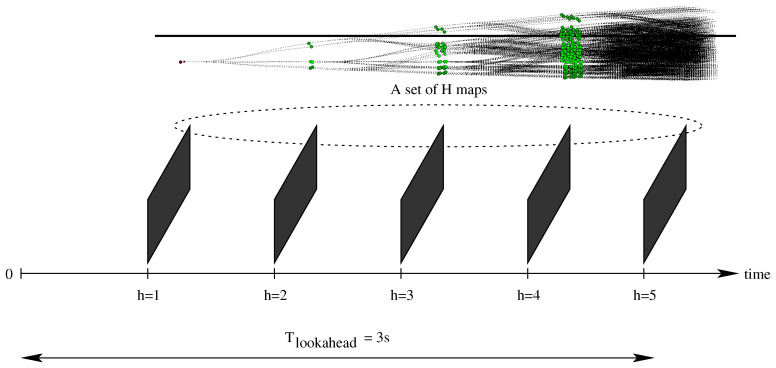
The synchronous communication scheme: The path planner runs once every time the data-fusion system generates a set of maps. The path-planner generates an expansion tree of depth 5, i.e., h∈[1,H]=[1,5], and spanning a total lookahead time of 3 s in the future. Consequently, the data-fusion system must generate, and transfer to the planner, a set of H=5 maps. These maps must represent the environment in the future every $T_{sim}=600$ ms, i.e., at $T_{0}$, $T=T_{0}+600$ ms, $T=T_{0}+1200$ ms, etc.

In the synchronous scheme, we set $T_{cycle}=T_{g}$. In our environment, we selected their value equal to 200 ms. As each path-planner execution estimates the path through the generation of an expansion tree of height *H*, and each tree level must use a proper map to control the trajectory of the car (and to evaluate the smaller trajectory cost), the fusion system must generate a set of *H* maps every 200 ms. Moreover, each one of these *H* maps, should represent the environment surrounding the car at increasing time point in the future. In fact, as described in Section 4.3, $T_{lookahead}=H\cdot T_{sim}$. Thus, if we set the simulation time $T_{sim}$ to 600 ms, and we consider expansion trees of height H=5, we have $T_{lookahead}=H\cdot T_{sim}=5\cdot 600$ ms = 3000 ms = 3 s. This implies that our *H* maps must represent the environment for 3 s into the future. The corresponding representation over time is reported in Figure 11.

The main advantage of this scheme is that the data-fusion system and the path planner is loosely coupled, and the synchronization effort is minimized. This reduces computational costs and memory usage. Unfortunately, the overall behaviors are not as reactive as desired in critical situation, as the local planner may definitely cycle faster and would benefit from more up-to-date maps.

### 6.2. Asynchronous Path Planner

In the asynchronous scheme, we maintain the map generation rate equal to $T_{g}=200$ ms, but to be sufficiently reactive, we make the planner work at 50 Hz, namely $T_{cycle}=20$ ms.

**Figure 12:**
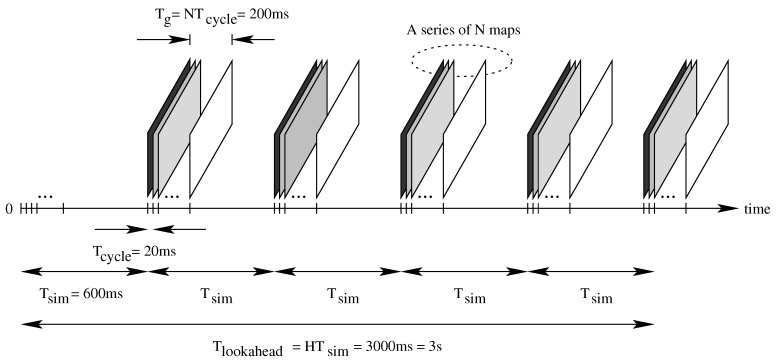
The asynchronous communication scheme: The path planner runs once every $T_{cycle}=20$ ms even if the data-fusion system generates a set of maps every $T_{g}=200$ ms. As in the synchronous case, during each run, the path planner generates an expansion tree of depth 5 (and spanning a total lookahead time of 3 s in the future). Unfortunately, the path planner runs N=200/20=10 times for every single data-fusion run. Consequently, the data-fusion system must generate, and transfer to the planner, a set of (N·H), i.e., 10·5=50, maps representing the environment. Maps belonging to the same tree level (a series of maps) have a time difference of 20 ms. Maps belonging to the same expansion tree (a set of maps) have a time difference of 600 ms.

Given these values, the data-fusion system must predict several maps sufficient for the planner to run $N=T_{g}/T_{cycle}$ times, namely N=200 ms/20 ms = 10 times. Moreover, as during each one of these *N* runs, the planner generates a tree with height *H*, the fusion system should generate (N·H) grid maps every $T_{g}$ seconds, i.e., *H* *series* of maps each one including a *set* of *N* maps. If H=5 and, as previously computed, and N=10, the fusion system should generates 50 grid maps every 200 ms. If we maintain a look-head time of $T_{lookahead}=3$ s, the overall situation is depicted by Figure 12. Each map must represent the environment around the car in the future at instant *T*

(1) $$\begin{array}{ccc} T & = & T_{0}+\left(h-1\right)\cdot T_{sim}+\left(n-1\right)\cdot T_{cycle} \end{array}$$

where $T_{0}$ is the current time (corresponding to the initial position of the vehicle), h∈[1,H] specifies the level of the expansion tree, and n∈[1,N] indicates the planner run within the set of runs belonging to the same window of grid-map generation. We consider all maps belonging to the same tree level (*h* fixed, *n* varies) a *series* of maps, and all maps belonging to the same expansion tree (*h* varies, *n* fixed) a *set* of maps.

Is this case the system is more reactive, but memory management is more critical. We will compare the synchronous and asynchronous strategies in Section 6.

## 7. Experimental Analysis

As represented in Figure 1 and discussed in all previous sections, our system put together several units and its behavior is influenced by several design options and implementation parameters. Map representation and the related path accuracy, communication protocol (synchronous or asynchronous), map transfer rate, object position estimation, and the different possible CPU-to-GPU data transfer schemes strongly influence the system and are the subjects of our experimental investigation. The theoretic paths (when there are no obstacles on the trajectory) or the ones generated by the CPU with the setting adopted by Cabodi et al. [7] (when there are obstacles on the trajectory) will be considered to be a reference. Our analysis evaluates the quality of the paths generated by the GPU-based concurrent version with the different settings. It will prove that the optimizations proposed in the previous sections not only do not deteriorate the quality of the generated paths but allow a higher reactivity and better vehicle safety.

In Section 7.1 we present our hardware configuration and in Section 7.2 we discuss the methods used to evaluate the quality of the generated trajectories. After that, we focus on the following operational scenarios. In Section 7.3, we will compare the synchronous and the asynchronous working modes. In Section 7.4, we will analyze the behavior of our system to avoid a collision with one or more static objects standing on the vehicle trajectory. In Section 7.5, we will concentrate on a back on track maneuver. Finally, in Section 7.6, we will present an analysis of the moose (or *elk*) test.

Please notice that as the system has been developed under an industrial non-disclosure agreement between Politecnico di Torino and Marelli, the final application, the benchmarks, and the set of experiments cannot be made available.

### 7.1. Experimental Set-Up

**Figure 13:**
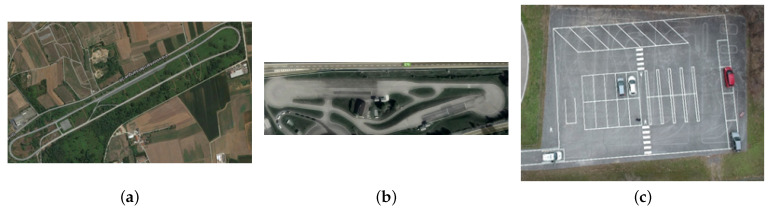
Private facilities adopted by the team of Automated Driving Technologies in Marelli to exercise their vehicles. (**a**) reports the highway installation, (**b**) the urban complex, and (**c**) the parking center.

To perform the necessary driving session the team of Automated Driving Technologies in Marelli resorts to some private facilities in which it is possible to reproduce real scenarios with proper speed limits and lane structure, and road signs as defined by the Italian road traffic regulation. These facilities cover usual traffic outlines and are reported in Figure 13:

- The highway center (Figure 13a) has three lanes, each one with a width included between 3.60 m and 3.75 m and a straight lane longer than 2 Km. As the speed limit is fixed at 130 km/h, this complex enables all scenarios possible on a highway as adaptive cruise control tests, overtaking, entering, exiting, and traffic jam. The standard level of automation in this installation is 3, with a driver on-board at all times.
- The urban facility (please see Figure 13b) has lanes with a width of 2.50 m and a speed limit of 50 km/h. This complex enables typical urban driving conditions as T-intersection, roundabouts, stop and go, traffic jam, and right of way intersections. The minimum level of automation needed in this complex is 3.
- The last installation (reported in Figure 13c) is a parking area facility with the speed limit settled at 15 Km/h. The area includes three different types of parking slots, i.e., parallel, orthogonal, and angular. This kind of scenario is used to test all conditions of a typical valet parking where the vehicle can park itself (level 4 of automation) without any driver or passenger inside.

In the experiments reported in the following sections, we set all conditions as reported in Table 1. All simulations are conducted considering a sensor configuration capable of identifying obstacles in a 140 m radius with a 1.5 s delay to process an obstacle from its appearance to its recognition. At all times, maneuvers are accomplished using a kinematic model compatible with the vehicle and the environment parameters. Suitable speeds for the experiments are computed using motion equations and the available vehicle models. The vehicle control system sends commands to the actuators at a fixed frequency rate, varying from 50 Hz to 100 Hz. The planner and the controller frequencies are either asynchronous or synchronous, following the schemes presented in Section 6.

Our planner runs on the following hardware devices:

- A CPU Intel Core i7-6700 HQ with 2.60 GHz and 8.00 GByte of RAM memory.
- A PX2 NVIDIA pascal GPU PG418 up to 3 GHz and 8.00 GByte DRAM per Parker.

### 7.2. Validation Methods

Usually, the way to quantify the accuracy of a model is by minimizing some error function that measures the misfit between the computed and the reference path. We will use several metrics to validate our paths. To define these metrics, we indicate with: $\left({\hat{x}}_{i},{\hat{y}}_{i}\right)$ the i-th point of the reference path, $\left(x_{1},y_{i}\right)$ the i-th path-planner-generated point, and with $n_{sample}$ the number of generated points. Based on these notations, the metrics that we will use are the following:

- The mean squared error (MSE) measures the average of the squares of the errors:
  (2) $$\begin{array}{ccc} \mathrm{MSE}(x,\hat{x},y,\hat{y})\; & = & \frac{1}{n_{\mathrm{samples}\;}}\cdot \sum_{i=0}^{n_{\mathrm{samples}\;}-1}\left[\left(x_{i}-{\hat{x}}_{i}^{2}\right)+\left(y_{i}-{\hat{y}}_{i}^{2}\right)\right] \end{array}$$
  It is the second moment (about the origin) of the error and thus incorporates the variance of the calibration curve:
- The Mean Bias Error (MBE) is usually adopted to capture the average bias in a prediction and it is defined as follow:
  (3) $$\begin{array}{ccc} \mathrm{MBE}(x,\hat{x},y,\hat{y})\; & = & \frac{1}{n_{\mathrm{samples}\;}}\cdot \sum_{i=0}^{n_{\mathrm{samples}\;}-1}\left[\left(x_{i}-{\hat{x}}_{i}\right)+\left(y_{i}-{\hat{y}}_{i}\right)\right]. \end{array}$$
- The root mean squared error (RMSE) compares data sets with different sizes, since it is measured on the same scale as the target value. It is obtained as the square root of the MSE, i.e.,
  (4) $$\begin{array}{ccc} \mathrm{RMSE}(y,\hat{y})\; & = & \sqrt{\mathrm{MSE}(y,\hat{y})}. \end{array}$$
  RMSE essentially measures the root of the mean square distance between the computed trajectory and the desired one. Usually, RMSE as to be as small as possible.
- The Centered Root Mean Square Error (CRMSE) is the RMSE corrected for bias. The CRMSE is defined as:
  (5) $$\begin{array}{ccc} CRMSE & = & RMSE\cdot sign(\sigma_{model}-\sigma_{reference}) \end{array}$$
  where σ is the standard deviation of the measure.

For these metrics we will apply the implementation available in the SciKitLearn Python library. Moreover, to deepen our analysis, we will also generate target diagrams [54]. In a target diagram, the x-axis indicates the CRMSE (computed as in Equation (5)) and the y-axis the MBE (please refer to Equation (3)), both normalized by the standard deviation of the reference $\sigma_{reference}$. Consequently, the vector distance between any given point and the origin is the RMSE normalized by the standard deviation of the reference measurements.

### 7.3. Comparing the Synchronous and the Asynchronous Mode

In this section, we compare the synchronous and the asynchronous modes introduced in Section 6 in terms of path accuracy.

**Figure 14:**
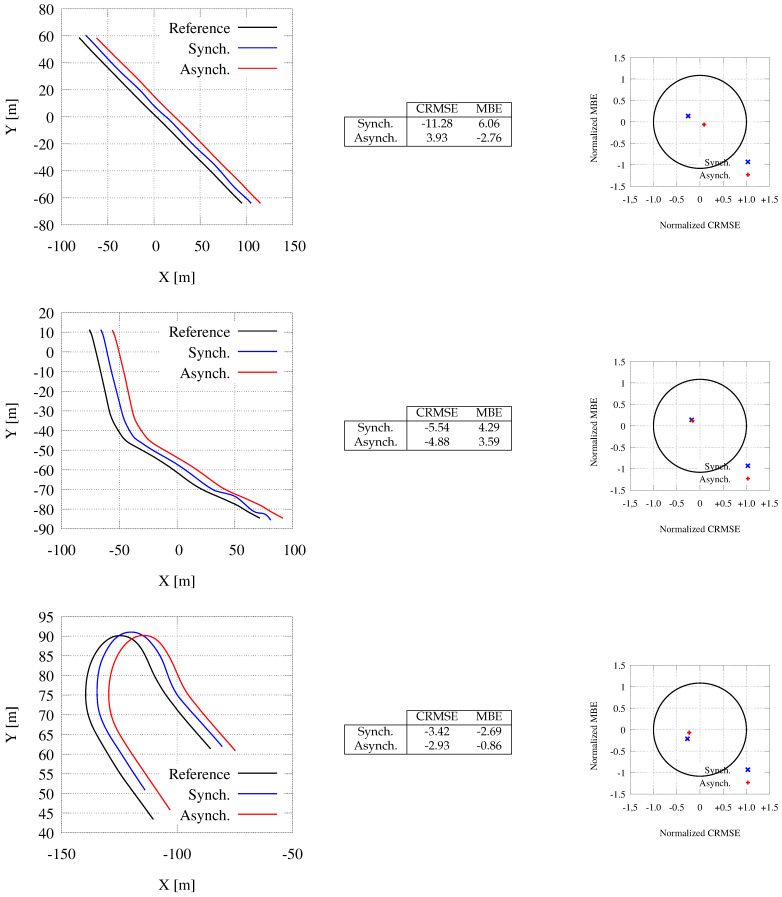
A comparison between the reference trajectory (the one to follow) and the paths generated using the synchronous and asynchronous communication schemes along a straight road (**top**), a mild bend (**center**), and a high curvature trajectory (**bottom**). The reference path and the ones gathered with synchronous and asynchronous working are displaced by 10 meters along the x-axis for readability. The error metrics (CRMSE and MBE), and target diagrams are reported in the center table and on the right-hand side plot.

Figure 14 presents experiments in three different scenarios: A straight trajectory (top), a low curvature path (i.e., a mild bend, middle), and a high curvature one (a roundabout, bottom) (Please be reminded that the curvature is a measure of how quickly a tangent line turns on a path.). For each of them, the figure reports: The reference path and the computed paths with both the synchronous and the asynchronous strategy (left-hand side), the values for the error metrics MBE and CRMSE (center), and the target diagrams (right-hand side). In each plot on the left-hand side, the 3 paths are displaced by 10 m along the x-axis to avoid an almost complete overlapping among them. The target diagrams show reasonable errors for all computed paths, as all points are within the unit circle. Anyway, both the plots and the error metrics show how the asynchronous communication strategy (with $T_{g}=200$ ms and $T_{cycle}=20$ ms) is far more accurate than the synchronous one (with $T_{g}=T_{cycle}=200$ ms) to follow the given paths. Thus, the points representing the asynchronous behavior are closer to the origin than the synchronous marks. Unexpectedly, the synchronous transfer mode present far larger errors for the straight path, representing the fact that even for very simple trajectories larger communication times can produce unexpected behavior. On the contrary, the asynchronous transfer mode allows very similar behavior in the 3 maneuvers and it guarantees consistent accuracy in all of them. Moreover, the on-board (and the simulator) analysis shows undesired behaviors that cannot be represented by the static plots. With the synchronous communication scheme, the vehicle proceeds at a higher speed and it shows undesired behavior with unsafe decelerations. In many cases, (please see the termination section of the blue path for the bend maneuver) the path follows an oscillatory trend with continuous path corrections, unsafe behaviors, and uncomfortable adjustments. These undesired phenomenon increase when we rise the map generation time $T_{g}$ beyond 200 ms. For example, with $T_{g}=500$ ms, the driver should retake control of the car in several situations to avoid collision, such as the end of the bend and at the end of the roundabout.

### 7.4. Obstacle Avoidance

For the obstacle avoidance case, we consider the same scenarios analyzed in the previous section. Anyway, for the sake of brevity, we report evidence only for the straight path. We analyze our tools with 3 different configurations. With Reference, we denote the CPU-based tool with the setting used by Cabodi et al. [7]. With Setting 1, we consider the GPU-based path planner with the same settings used for the CPU. With Setting 2, we refer to the GPU-based path planner with the strategies used to manipulate and transfer the grid maps introduced in Section 3.3. More specifically, we:

- Reduced the grid-map resolution to (500×500) pixels and we displaced the car such that the map in front of the vehicle represents a larger area than the one spanned behind it.
- Represented the probability of each location of each grid map with a reduced accuracy, i.e., using a short integer for each location.
- Merged all maps within the same map series into the same grid map.
- Used textures memory to represent from 4 to 16 different grid maps eventually belonging to the same series of maps or to the same set of maps.
- Used the same grid map computed for the tree level h=4, for each further computation level with h>4.

In all cases, we consider a lookahead time equal to $T_{lookahead}=3000$ ms = 3 s, which represents a good trade-off between accuracy and computation costs. We analyze our tool with one or two obstacles statically lying directly on the vehicle straight path.

**Figure 15:**
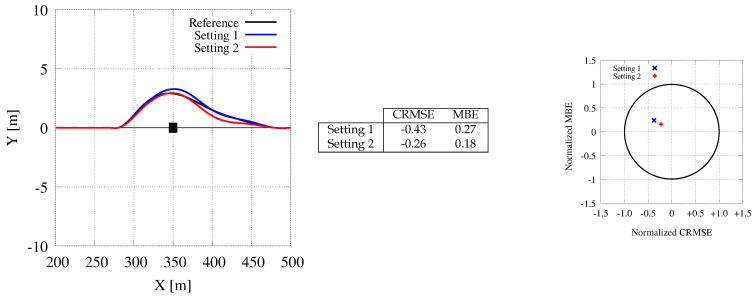
Generated paths for a scenario of a single obstacle avoidance. The CPU trajectory with the setting used by Cabodi et al. [7] (black color) is represented as a reference for the GPU ones (blue and read colors). The two GPU trajectories are computed with different settings.

Figure 15 concentrates on one single obstacle, with the car moving at a speed of 20 m/s (i.e., 72 Km/h). The differences in the computed paths are minimal, and mainly due to different approximations performed by the CPU and the GPU during the computation. Moreover, computational differences are mainly limited to the initial part of the maneuver, i.e., while the computational constraints are tighter and the system must be more reactive. The ending part of the trajectories are mainly influenced by these initial choices. As proved by the error metrics (MBE and CMRSE) and the target diagram, the differences are negligible, and the approximation strategies adopted to save memory space and computation time are effective and do not deteriorate the paths evaluated by the tool.

**Figure 16:**
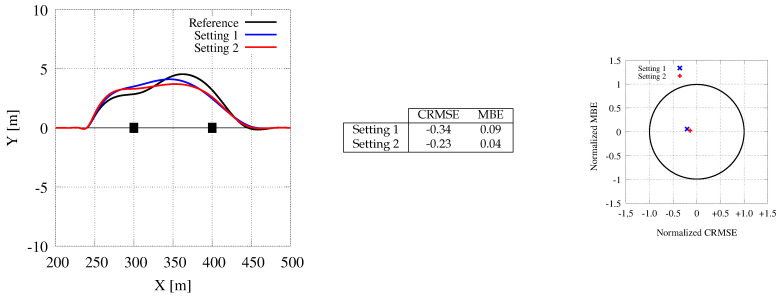
Generated paths for a scenario of a double obstacle avoidance. The CPU trajectory with the setting used by Cabodi et al. [7] (black color) is represented as a reference for the GPU ones (blue and read colors). Notice that in this case, the CPU trajectory is riskier than the ones computed with the GPU as it gets too close to the first obstacle due to slower computation times.

Figure 16 shows the paths computed with two obstacles. In this case, the GPU is faster to react to the first obstacle. Thus, both paths computed by the GPU (with Setting 1 and Setting 2) are faster to move away from the first obstacle and then they maintain a quite linear behavior. On the contrary, the CPU is slower to anticipate the new path and somehow it must recover from this slowness once the second obstacle has been spotted. Thus, the trajectory requires further adjustments moving from the first to the second obstacle. Conclusions are similar to the ones reported for the previous case when we compare the two GPU paths, with minor discrepancies between them and small errors compared to the reference one.

### 7.5. Back on Track

For the back on track analysis, we run the experiment with the car starting at a certain distance (that is, 20 m) from the desired path and converging toward it as fast as possible. As in the previous sections, we analyzed 3 scenarios, considering the back on track maneuver on a straight, a low curvature, and a high curvature path. Anyway, for the sake of brevity, we report evidence only for the first scenario. When the path is straight, moving back on track can be seen as a lane change maneuver or as a generalization of the path following maneuver. The experimental setting is the one specified in Section 7.4.

**Figure 17:**
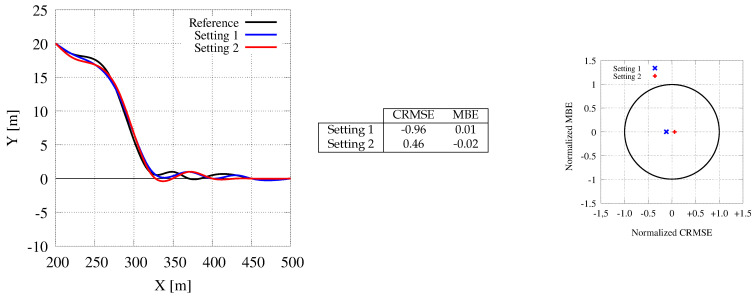
Generated paths for a back on track maneuver. The CPU trajectory (black color) is represented as a reference for the GPU one (blue and red colors, with Setting 1 and 2).

Figure 17 shows the actual paths followed during this experiment by the CPU-based and the GPU-based tool versions (the latter with two different settings). Again, differences on the generated path are limited to small discrepancies in the initial part of the maneuver, with a subsequent impact on the remaining part where a sort of harmonic trajectory is used to move back on the straight path. The plot also shows that (again) the GPU-based tool is faster to react (i.e., to close the gap with the target path at the beginning of the maneuver) and that our optimizations (to reduce memory and communication costs) do not deteriorate the quality of the path followed by the car. Indeed, the target diagrams show points very closed to the origin, i.e., related to paths with an extremely small normalized RMSE.

### 7.6. The Moose or elk Test

In the moose (or *elk*) test, the vehicle must dodge two obstacles, the first one on the same lane and the second one on the fast late. Forms of this test have been performed in Sweden since the 1970s. It has been standardized in ISO 3888-2, and it is usually performed to determine how well a certain vehicle evades a suddenly appearing obstacle. With a moose test, we can also simulate a sequence of path followings, a lane changes, and two obstacle avoidance at the same time. Moreover, this test can also be seen as a vehicle overtaking or as a a sequence of two vehicle obstacle avoidance.

**Figure 18:**
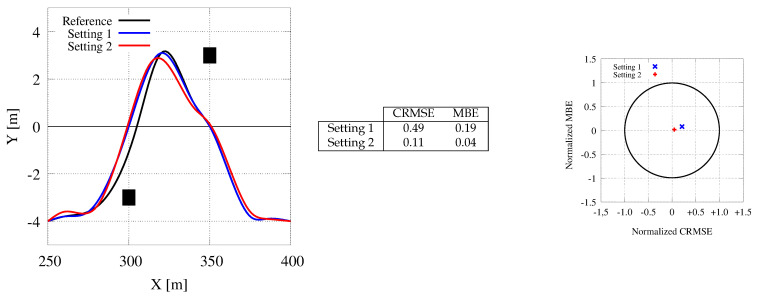
Generated paths for the elk (or moose) maneuver. The CPU trajectory (black color) is represented as a reference for the two GPU ones (blue and red colors).

Figure 18 reports the paths gathered for the elk test maneuver with our CPU-based and GPU-based applications (the latter one again with two different settings). The distance between the two obstacles is set to 50 m on the x-axis and to 5 m on the y-axis. The reference path is at y=0 m, and the vehicle starts simulation at y=−4 m at the center of the right lane. Grid maps limit the road from y=−6 m to y=+6 m. The remaining settings are the one used in the previous experiments. Our analysis leads to the same considerations presented before. The plot shows that the GPU-based tool is faster to react to the first obstacle and that our memory and communication cost reduction do not deteriorate the quality of the path followed by the car. Again, the target diagrams show trajectories with very small normalized RMSE.

## 8. Conclusions

Autonomous vehicles, for both personal and freight transportation, potentially offer huge enhancements to society, business, and every-day life. Most of the automatic driving systems divide their massive task into sub-tasks, assign each sub-task to a different module, and employ different techniques, strategies, and algorithms within each module.

In our project, which began in 2017, we first focused on the path planner. Starting from the work by Schwesinger et al. [6], we developed a CUDA-based GPGPU implementation of a randomized sampling-based motion planning strategy. We describe that task in [7], detailing the implementation to find safe collision-free trajectories and proving the computational efficiency of our solution. In the current work, we focus on how the planner module has been inserted into a complete and working loop chain. The first module of our chain is the sensor module which must collect a huge amount of information related to the environment. In this area, we describe the advantages and disadvantages of the different sensors and we select the right type and number of sensors for our target, finally employing long-range and medium-range radars, lidars, and cameras. The second module is the data-fusion unit, which combines all information into a proper grid-based representation of the reality. In this section, we show how it is possible to play with spatial resolution, object enlargement, and a few other parameters, to reduce memory occupation and communication costs between the different modules. At the path planning level, we mainly concentrate on communication efficiency and cost. We present a synchronous communication scheme in which the path planner is tightly synchronized with the data-fusion system, as it runs only once every time a new map set is ready to be used. This scheme, albeit simple, shows reduced reaction times, especially in critical conditions. Then, we present an asynchronous communication scheme in which the path planner runs at a higher rate. In this case, the communication scheme is not trivial and many grid maps must be deduced at the same time to forecast the path and the vehicle’s behavior in future computational cycles. We show that this scheme is more complex than the previous one but it is also more reactive, and it guarantees better behavior in all safety-critical conditions. Finally, we present our controller unit, which is derived from off-the-shelf components and applications.

In the experimental result section, we show the behavior of our concurrent implementation on different scenarios and with different settings. We compare our GPU-based implementation with the original CPU-based one, in terms of standard error metrics. Our results show a higher reactivity, better safety, and a very good path accuracy. Overall, the work done to develop the required modules, the effort to put them together, and their validation on the road, constitute the main contributions of this work.

## Figures and Tables

**Table 1:** Vehicle characteristics and driving conditions in the set of experiments described in the following subsections.

Vehicle Characteristics	Values
Width	2 m
Maximum lateral offset	0.8 m
Speed	[13 m/s, 36 m/s]
	[46.8 Km/h, 29.6 Km/h]
Acceleration (standard)	[−2 m/s${}^{2}$, +2 m/s${}^{2}$]
Acceleration (emergency)	[−9.81 m/s${}^{2}$, +9.81 m/s${}^{2}$]
